# Leiomodin 1 deficiency promotes lipid accumulation and redirects gene regulatory programs in smooth muscle cells exposed to oxidized LDL

**DOI:** 10.21203/rs.3.rs-10473063/v1

**Published:** 2026-08-26

**Authors:** Sara A. Wennersten, Hongxia Wang, J. Lee Franklin, Vivek Nanda

**Affiliations:** University of Alabama at Birmingham

**Keywords:** leiomodin 1, vascular smooth muscle cells, gene regulatory networks, lipid accumulation, foam cell-like cells, phenotypic modulation

## Abstract

The transition of smooth muscle cells (SMCs) from a contractile to a synthetic, lipid-accumulating phenotype is a central driver of atherosclerosis. We previously identified leiomodin 1 (*LMOD1*), an SMC-enriched gene, as a critical regulator of SMC phenotypic modulation and atherosclerosis in mice. However, whether *LMOD1* exerts similar effects in human SMCs, and the mechanisms underlying its anti-atherogenic effects, remain unclear. Here, we show that *LMOD1*-deficient human SMCs exposed to oxidized low-density lipoprotein (oxLDL) exhibit increased intracellular lipid accumulation, consistent with a foam cell-like phenotype. Bulk RNA-sequencing using a Condition × Treatment interaction-term design identified *LMOD1*-dependent oxLDL-responsive transcriptional changes, marked by altered atherogenic gene expression, including dysregulated *LDLR* and *BMP2* responses. Integrative regulatory network inference revealed that *LMOD1* deficiency disrupted the coordinated oxLDL-induced gene-targeting program observed in control SMCs. Response-vector geometry further demonstrated that *LMOD1* loss redirected the regulatory response to oxLDL along a near-orthogonal axis in gene-targeting space. Interaction-term targeting and condition-specific propagation analyses uncovered widespread target-gene rewiring and altered transcription factor influence, including *FOXO1*- and *RUNX2*-associated inferred regulatory routes. Together, these findings provide a potential mechanistic basis for the anti-atherogenic effects of *LMOD1* in human SMCs.

## INTRODUCTION

Cardiovascular disease (CVD) remains the leading cause of death worldwide and continues to impose a growing burden despite advances in prevention and therapy[1–3]. Most CVD morbidity and mortality stem from atherosclerosis, a progressive accumulation of lipid-rich lesions within the arterial wall that underlies the majority of myocardial infarctions and strokes and is driven by both disordered lipid homeostasis and chronic vascular inflammation[4]. Current insights into the biology of atherosclerosis implicate the involvement of a heterogeneous population of cell types, including endothelial cells, leukocytes, macrophages, and vascular smooth muscle cells (SMCs)[5]. Notably, recent evidence indicates that the accumulation of SMCs within atherosclerotic lesions is a critical driver of increased lesion size, as these cells contribute substantially to plaque cellularity and adopt foam cell- and macrophage-like states[6–10].

SMCs localized within the medial layer of blood vessel walls are highly specialized, differentiated cells responsible for maintaining vascular wall integrity and regulating contraction[11]. In disease conditions such as atherosclerosis, however, SMCs display remarkable plasticity, transitioning from a quiescent, differentiated, contractile state to a dedifferentiated, synthetic phenotype[6,12]. This phenotypic switch is characterized by the downregulation of several key SMC contractile marker genes, including transgelin (*TAGLN*), myocardin (*MYOCD*), and myosin heavy chain 11 (*MYH11*)[11,13–16]. Emerging evidence indicates that within atherosclerotic lesions, SMCs transdifferentiate into macrophage-like cells, characterized by reduced expression of contractile genes, increased expression of macrophage-associated genes, and enhanced capacity for lipid uptake, a phenotypic adaptation implicated in the pathogenesis and progression of atherosclerosis[6–8,17,18]. Furthermore, restoring the expression of select SMC contractile genes has been shown to promote a more differentiated SMC phenotype and reduce phenotypic modulation, offering a potential platform for developing new therapeutic strategies against atherosclerosis[16,19–21]. However, despite these advances, the differential expression pattern of SMC contractile genes in atherosclerosis remains incompletely defined.

Recent studies, including our own, identified leiomodin 1 (*LMOD1*), an SMC-enriched gene critical for maintaining SMC contractile function, to be significantly downregulated in atherosclerosis[22–26]. Subsequently, loss of *Lmod1* was shown to promote the transition of SMCs to a foam cell-like phenotype and accelerate atherosclerosis in mice[24]. However, whether these effects are conserved in humans and the underlying mechanisms remain unclear. Here, we address this knowledge gap by modeling an atherogenic milieu *in vitro* and demonstrate, for the first time in primary cultured human SMCs, that *LMOD1* deficiency drives SMCs to transdifferentiate from a contractile to a foam cell-like phenotype, as evidenced by increased uptake of oxidized low-density lipoprotein (oxLDL). Transcriptomic analysis identified *LMOD1*-dependent oxLDL transcriptional changes involving inflammatory, lipid-metabolic, and SMC phenotypic modulation gene programs, including altered *LDLR* and *BMP2* responses. At the network level, *LMOD1* deficiency disrupted the coordinated gene-targeting gains and losses normally induced by oxLDL in control SMCs while redirecting the overall response toward a distinct regulatory state. Condition-specific transcription factor (TF) influence and propagation analyses further identified altered downstream regulatory routes, including distinct *RUNX2*- and *FOXO1*-associated programs. Together, these findings establish *LMOD1* as a key determinant of SMC fate under atherogenic stress. Because its loss drives lipid accumulation and extensive transcriptional and regulatory remodeling in response to oxLDL, maintaining *LMOD1* expression may represent a potential therapeutic strategy to limit SMC-derived foam cell-like formation and slow atherosclerotic plaque progression.

## RESULTS

### 1. LMOD1 knockdown exacerbates oxLDL-induced lipid uptake in SMCs

**Figure 1:**
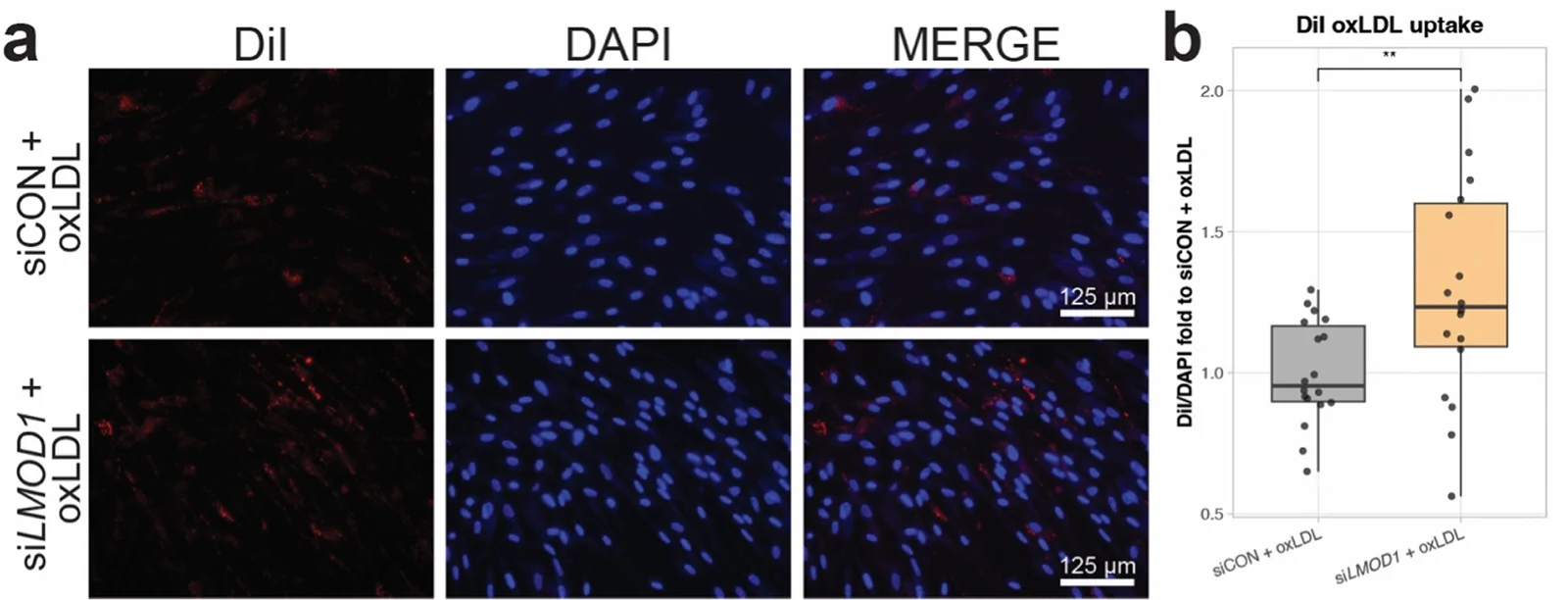
Reduced *LMOD1* enhances lipid uptake in SMCs. **(a)** Representative fluorescence images of cultured SMCs following exposure to DiI-labeled oxLDL (DiI-oxLDL). Compared to control-transfected cells (siCON + oxLDL), *LMOD1*-deficient SMCs displayed increased lipid uptake (red). **(b)** Quantification of DiI-oxLDL uptake expressed as a ratio of DiI fluorescence intensity (red) to DAPI-positive cell number (blue). Data are pooled from three independent experiments and presented as normalized fold change to siCON + oxLDL (siCON + oxLDL set to 1). **p < 0.01. Scale bars indicate 125 μm.

We recently demonstrated that SMC-specific deletion of *Lmod1* increased lipid uptake and accelerated atherosclerosis in mice[24]. To determine whether these effects are conserved in humans, we evaluated the impact of reduced *LMOD1* expression in cultured SMCs. Following confirmation of efficient *LMOD1* knockdown (Supplementary Figure S1), we induced an atherogenic stimulus *in vitro* by treating SMCs with DiI-labeled oxidized LDL (oxLDL) and compared lipid uptake between control (siCON) and *LMOD1*-deficient (si*LMOD1*) cells. *LMOD1*-deficient cells exhibited greater lipid accumulation than control cells, as evidenced by increased intracellular fluorescence (Fig. 1a) and quantitative analyses of fluorescence intensity (Fig. 1b; *p* = 0.0085). These findings reveal that reduced *LMOD1* expression enhances lipid uptake in SMCs, thereby promoting their transition toward a foam cell-like phenotype.

### 2. LMOD1 deficiency reprograms the oxLDL transcriptional response toward inflammatory, lipid-handling, and atherogenic gene programs

**Figure 2:**
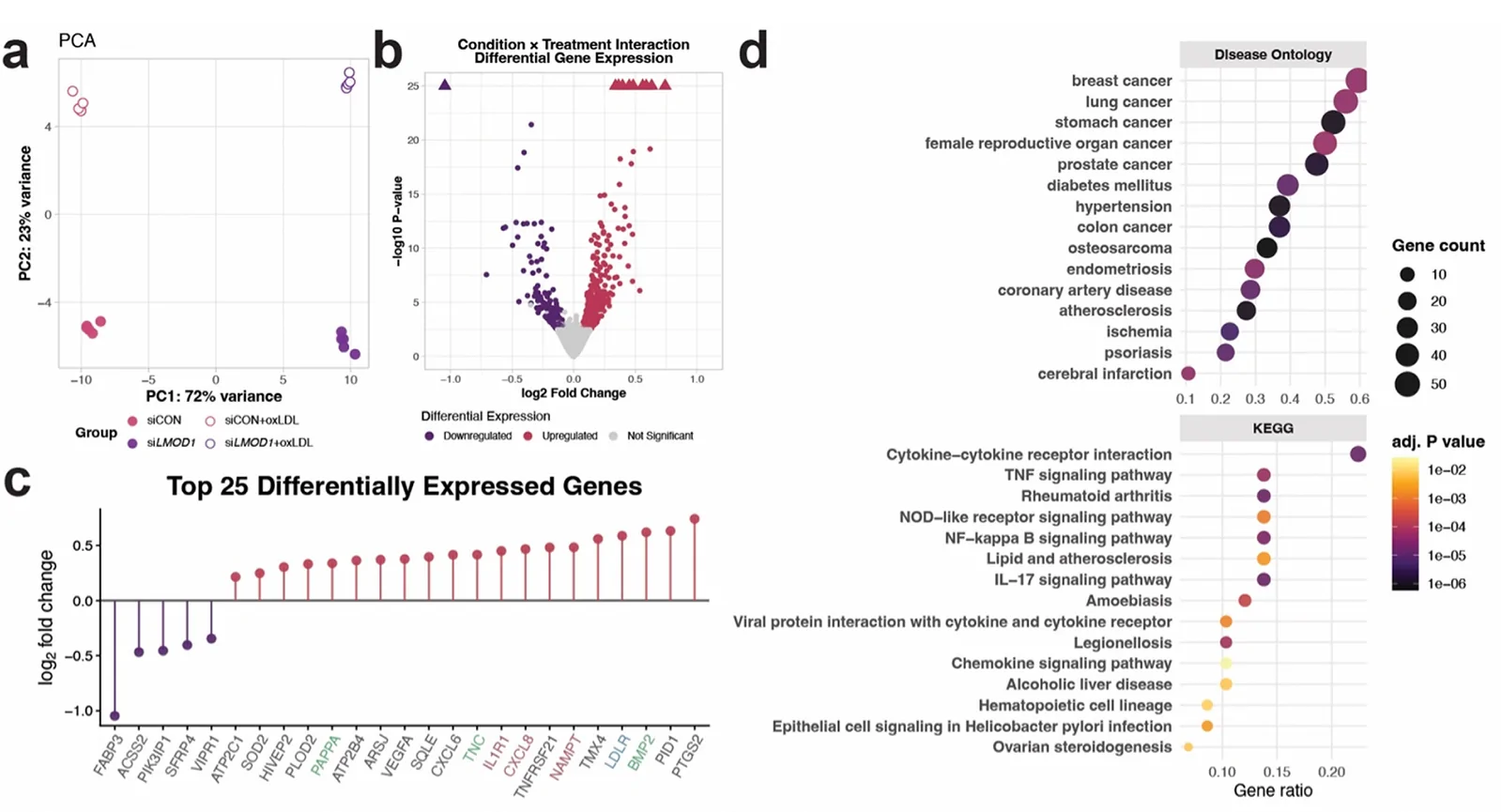
Transcriptomic changes and functional enrichment analysis in *LMOD1*-deficient SMCs treated with oxLDL. **(a)** Principal component analysis (PCA) of RNA-sequencing data showing separation of siCON (n=5), si*LMOD1*(n=5), siCON + oxLDL (n=5), and si*LMOD1* + oxLDL (n=5) groups. **(b)** Volcano plot illustrating differential gene expression for the Condition × Treatment interaction term (design = ~Condition × Treatment). The x-axis represents log2FC, and the y-axis represents −log10 *p*-value. Significantly differentially expressed up- or downregulated genes regulated (adjusted *p*-value < 0.05) are color-coded. Triangles indicate values beyond the axis limits. **(c)** Lollipop plot showing the top 25 differentially expressed genes associated with the Condition × Treatment interaction, ranked by adjusted *p*-value and displayed by log2FC. Each dot represents the estimated log2FC. Genes with a positive log2 FC are upregulated (pink), whereas genes with a negative log2FC are downregulated (purple). Gene labels are color-coded by SMC-supported functional association: inflammatory/cytokine signaling (red), extracellular matrix remodeling/SMC phenotypic modulation (green), or lipid-handling/oxLDL-response (blue). **(d)** Functional enrichment analysis of interaction-significant genes (|log2FC| > 0.25, adjusted *p*-value < 0.05). Dot plots display the top enriched terms from Disease Ontology and KEGG pathway analyses. The x-axis represents GeneRatio, dot size corresponds to the number of genes associated with each term; and color indicates the adjusted *p*-value.

To gain insight into the molecular mechanism linking *LMOD1* deficiency with increased lipid uptake, we performed RNA sequencing on control and *LMOD1*-deficient SMCs treated with or without oxLDL. Principal component analysis (PCA) showed clear separation among the four experimental groups (Fig. 2a). To identify genes whose transcriptional response to oxLDL was specifically dependent on *LMOD1*, we fit an interaction model and identified genes with significant *LMOD1*-oxLDL interaction effects. This analysis revealed widespread interaction-dependent differential gene expression (adjusted *p*-value < 0.05; Fig. 2b), indicating that *LMOD1* deficiency both enhanced and reduced oxLDL-induced transcriptional responses relative to siCON. Among the top 25 interaction-associated differentially expressed genes (Fig. 2c), color-coded gene labels highlighted functional categories related to SMC-relevant inflammatory/cytokine signaling (red), extracellular matrix remodeling/SMC phenotypic modulation (green), and lipid-handling/oxLDL-response genes (blue), consistent with coordinated transcriptional programs relevant to SMC phenotypic modulation during atherosclerosis[27–31].

Combined Disease Ontology and KEGG (Kyoto Encyclopedia of Genes and Genomes) pathway enrichment analyses of the interaction-significant gene set, visualized as a dot plot (Fig. 2d), reinforced this dual inflammatory- and lipid-associated transcriptional signature. The most significantly enriched Disease Ontology terms spanned a broad neoplastic spectrum (breast, lung, stomach, prostate, colon, and female reproductive organ cancers; osteosarcoma) as well as cardiovascular and metabolic disorders relevant to the observed foam cell-like phenotype, including coronary artery disease, atherosclerosis, ischemia, cerebral infarction, hypertension, and diabetes mellitus. KEGG pathway enrichment was dominated by innate immune and cytokine signaling pathways, including cytokine-cytokine receptor interaction, TNF, NF-κB, IL-17, NOD-like receptor, and chemokine signaling pathways, together with the “Lipid and atherosclerosis” pathway. Taken together, these findings reveal inflammatory signaling and lipid metabolism-related gene pathways as major transcriptional programs dysregulated by *LMOD1* deficiency during the SMC response to oxLDL.

### 3. Top differentially expressed genes suggest LMOD1 knockdown sensitizes SMCs to oxLDL-induced rewiring of lipid metabolism gene programs

**Figure 3:**
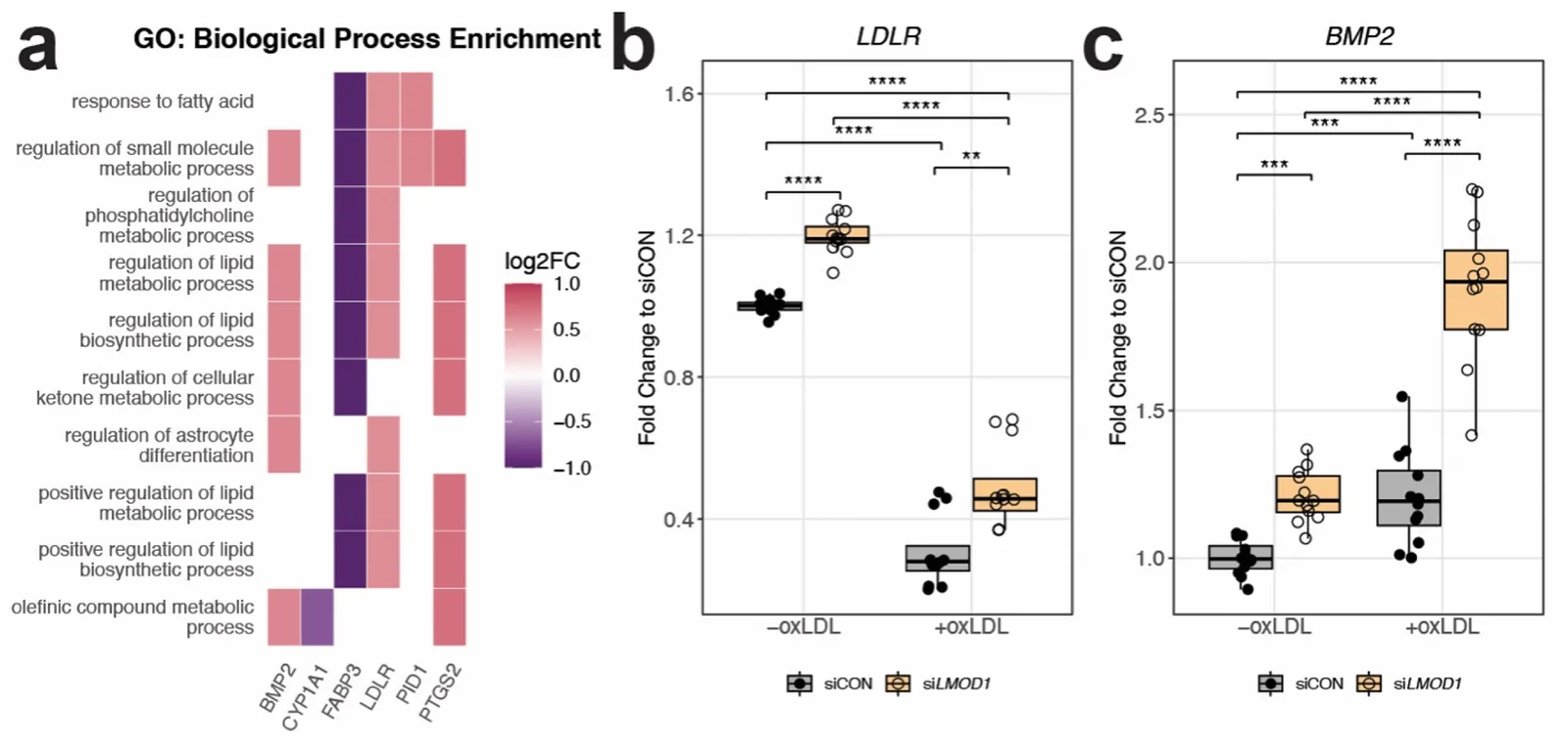
*LMOD1* knockdown sensitizes SMCs to oxLDL-induced rewiring of lipid metabolism. **(a)** Heatplot of the top 10 enriched Gene Ontology Biological Process (GO:BP) terms derived from interaction-significant differentially expressed genes (|log2FC| > 0.5, adjusted *p*-value < 0.05), generated using the heatplot function from the clusterProfiler R package. Rows represent enriched GO:BP terms and columns represent the genes contributing to each term. Cell color reflects the log2FC of each gene. **(b-c)** Quantitative RT-PCR validation of selected interaction-associated genes identified by RNA-seq. **(b)** *LDLR* expression is increased by *LMOD1* knockdown at baseline. Although oxLDL suppresses *LDLR* expression in both siCON and si*LMOD1* SMCs, *LDLR* levels remain significantly higher in si*LMOD1* + oxLDL cells compared to siCON + oxLDL cells. **(c)** *BMP2* expression is selectively upregulated under combined si*LMOD1* and oxLDL treatment. Data are pooled from three independent experiments and presented as box and whisker plots showing median, the interquartile range (25^th^-75^th^ percentiles) and whiskers extending to 1.5 × IQR. Individual data points are overlaid. Because normality and/or homogeneity of variance assumptions were not met, group differences were assessed using a Kruskal-Wallis test followed by pairwise Wilcoxon rank-sum tests with BH correction for multiple comparisons; **p < 0.01, ***p < 0.001, ****p < 0.0001.

To identify the biological processes most strongly altered by the *LMOD1* × oxLDL interaction, we performed Gene Ontology Biological Process (GO:BP) enrichment on interaction-significant differentially expressed genes filtered at |log2 fold change (log2FC)| > 0.5. The top 10 enriched terms (Fig. 3a) were strongly enriched for lipid and small-molecule metabolic processes. Of the genes contributing to these terms (*BMP2*, *CYP1A1*, *FABP3*, *LDLR*, *PID1*, and *PTGS2*), most were predominantly upregulated under combined *LMOD1* loss and oxLDL exposure, with the exception of *FABP3*, an intracellular fatty acid carrier, and *CYP1A1*, a cytochrome P450 involved in xenobiotic and lipid oxidation[32,33]. Together, this pattern suggests that *LMOD1* deficiency modifies lipid-handling programs in SMCs in response to oxLDL.

To validate the strongest interaction-specific differentially expressed genes identified by RNA-seq, we examined mRNA expression by qPCR across all four experimental conditions. Robust knockdown of *LMOD1* was confirmed in si*LMOD1*-transfected SMCs (Supplementary Figure S1). We then assessed two top-ranked candidates from the enrichment analysis: *LDLR* (Fig. 3b) and *BMP2* (Fig. 3c). Interestingly, *LDLR* mRNA was increased following *LMOD1* knockdown under basal conditions relative to control cells (siCON). In siCON SMCs, treatment with oxLDL reduced *LDLR* expression, consistent with canonical SREBP-mediated feedback regulation[27]. Although oxLDL also reduced *LDLR* expression in si*LMOD1* cells, *LDLR* levels remained significantly higher than in oxLDL-treated siCON cells, indicating that *LMOD1* knockdown alters the magnitude of the *LDLR* response to oxLDL (Fig. 3b). *BMP2* displayed a distinct interaction pattern, with modest increases following either *LMOD1* knockdown or oxLDL alone relative to siCON cells, and a marked increase under combined si*LMOD1* + oxLDL conditions (Fig. 3c), consistent with the RNA-seq interaction signal (Fig. 2c). Together, these findings support the condition-specific *LDLR* and *BMP2* expression patterns predicted by the RNA-seq interaction analysis.

### 4. LMOD1 deficiency disrupts coordinated oxLDL-induced gene targeting programs and redirects the network response

**Figure 4:**
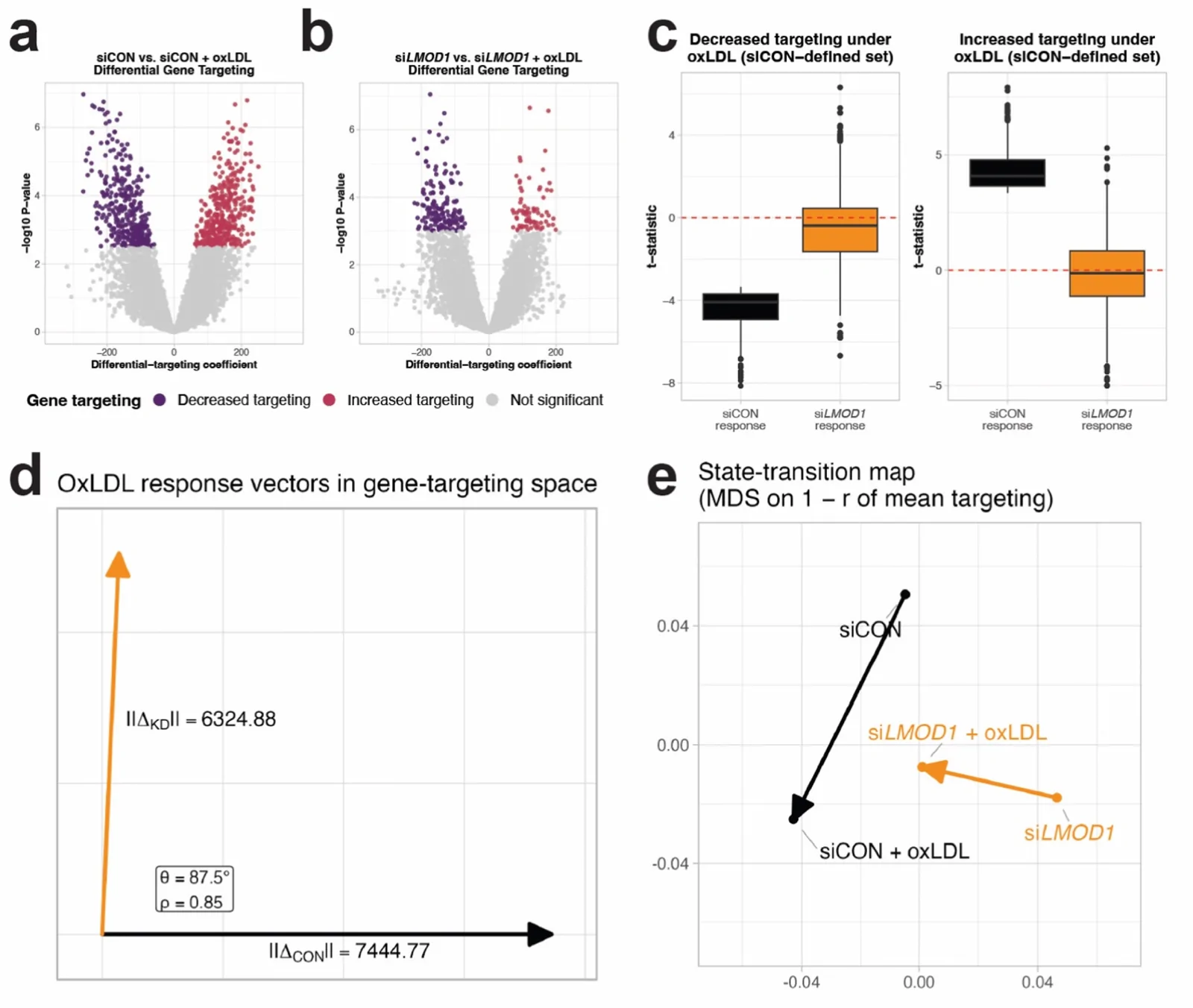
Inferred gene regulatory networks reveal altered oxLDL-induced gene targeting responses in *LMOD1*-deficient SMCs. Volcano plots showing differences in gene targeting between **(a)** siCON and siCON + oxLDL and **(b)** si*LMOD1* and si*LMOD1* + oxLDL, based on gene-targeting scores derived from sample-specific LIONESS networks generated using PANDA-integrated regulatory networks (analysis performed using limma with BH-adjusted *p*-values). Genes with significantly increased targeting under oxLDL are pink; significantly decreased are purple; non-significant are gray (adjusted *p*-value < 0.05). **(c)** Paired comparison of siCON-defined increased- and decreased-targeting gene sets. Left, genes with decreased targeting under oxLDL (paired Wilcoxon signed-rank, siCON < si*LMOD1*): *n* = 387, V = 361, *p* < 2.2 ´ 10^−16^; matched-pairs rank-biserial 𝑟=0.858 (95% CI 0.85–0.86). Right, genes with increased targeting under oxLDL (paired Wilcoxon signed-rank, siCON > si*LMOD1*): n = 419, V = 87,924, *p* < 2.2 ´ 10^−16^; matched-pairs rank-biserial 𝑟 = 0.865 (95% CI 0.86–0.87). Median *t*-statistics shift toward zero in si*LMOD1*, indicating that the coordinated siCON targeting response is not reproduced following *LMOD1* knockdown. **(d)** OxLDL response vectors in gene-targeting space. Arrows originate at baseline and terminate at the treatment-induced shift (Δ) for each genotype, where Δ_CON_ represents the siCON ^®^ siCON + oxLDL response and Δ_KD_ represents the si*LMOD1* ^®^ si*LMOD1* + oxLDL response. Arrow length reflects the overall magnitude of the targeting response, and the angle θ between vectors quantifies directional similarity of the oxLDL responses. The near-orthogonal angle (θ = 87.5°) indicates that the *LMOD1* knockdown response is redirected relative to the control response rather than simple attenuation or inversion. **(e)** State-transition map generated by classical multidimensional scaling (MDS) on the disparity matrix (1 − *r* of mean gene targeting), where each point represents one condition and arrows trace the baseline ^®^ oxLDL transition within each genotype (siCON ^®^ siCON + oxLDL in black; si*LMOD1* ^®^ si*LMOD1* + oxLDL in orange). Inter-point distances reflect endpoint dissimilarity in gene-targeting profiles. The control and knockdown transitions occupy distinct trajectories in the MDS embedding, consistent with the vector analysis shown in **(d)**.

Because SMC-specific phenotypic shifts can arise through coordinated regulatory rewiring without substantial changes in individual transcript levels, conventional differential expression analyses may not fully capture the effects of *LMOD1* deficiency[34–36]. We therefore implemented a network-based framework to identify coordinated alterations in transcriptional regulation induced by *LMOD1* deficiency under lipid stress. Specifically, we inferred sample-specific gene regulatory networks (GRNs) with PANDA (Passing Attributes between Networks for Data Assimilation) and LIONESS (Linear Interpolation to Obtain Network Estimates for Single Samples) by integrating TF-motif, protein-protein interaction (PPI), and gene expression priors[37,38]. We then quantified each gene’s aggregate regulatory input as its in-degree (the sum of incoming TF→gene edge weights), hereafter referred to as gene targeting[36]. In control SMCs, oxLDL treatment elicited a broad, bidirectional shift in targeting (Fig. 4a). Numerous genes significantly gained (pink) or lost (purple) incoming regulatory influence, suggesting a coordinated reallocation of TF activity in response to lipid stress. In contrast, *LMOD1*-deficient (si*LMOD1*) SMCs showed a reduced number of genes with significant targeting changes (Fig. 4b), suggesting a dampened regulatory response to oxLDL. We next sought to assess the oxLDL response at three levels: the preservation of individual gene-targeting programs across genotypes, the overall geometry of the network-wide response, and the separation among condition-level regulatory states.

To compare preservation of the siCON-defined oxLDL-induced gene-targeting programs across genotypes, we adapted an approach described by Saha et al.: we extracted per-gene *t*-statistics for the significant siCON-defined increased- and decreased-targeting gene sets and compared them with the corresponding per-gene *t*-statistics from the si*LMOD1* contrast (Fig. 4c)[39]. Paired, gene-matched Wilcoxon signed-rank tests showed that, for both the decreased- and increased-targeting gene sets, the oxLDL-induced targeting shifts in si*LMOD1* cells were significantly closer to zero relative to siCON SMCs, indicating that the coordinated oxLDL-induced targeting changes observed in control cells were largely not reproduced after *LMOD1* knockdown (decreased-targeting set, siCON < si*LMOD1* (n = 387, V = 361, *p* < 2.2 × 10^−16^; rank-biserial *r* = 0.858, 95% confidence interval (CI) 0.85–0.86); increased-targeting set, siCON > si*LMOD1* (n = 419, V = 87,924, *p* < 2.2 × 10^−16^; *r* = 0.865, 95% CI 0.86–0.87; Fig. 4c)). Because these gene sets were defined by significance in the siCON contrast, this paired comparison is descriptive and prone to regression to the mean. We therefore treat Fig. 4c as a set-level summary supported by the selection-independent Correlation Adjusted MEan RAnk (CAMERA) test, which accounts for inter-gene correlation. As expected, the siCON-defined sets were significantly enriched in the corresponding siCON contrast (Inc: Up, FDR = 2.83 × 10^−4^; Dec: Down, FDR = 2.83 × 10^−4^; Supplementary Table S1). Given that these same data were used to define the gene sets, this result serves as a positive control rather than independent confirmation. The informative, non-circular test is whether these siCON-defined gene sets are also enriched in si*LMOD1* SMCs. In si*LMOD1* cells the decreased-targeting set remained directionally down but was not significantly enriched (Dec: Down, FDR = 0.710) and the increased-targeting set was also not significantly enriched and instead trended in the opposite direction (Inc: Down, FDR = 0.866).

Whereas the gene-set analysis in Fig. 4c tested preservation of siCON-defined targeting programs rather than the total size of the network response, we next examined the oxLDL response at the global network level by constructing an oxLDL response vector (Δ) for each genotype by subtracting the mean baseline targeting profile from the mean oxLDL-treated profile and then comparing the magnitudes of these vectors and the angle between them. The knockdown response vector (‖Δ_KD_‖ = 6,324.88) was shorter than the control response vector (‖Δ_CON_‖ = 7,444.77; raw attenuation ratio of 0.85; Fig. 4d, Supplementary Table S1). However, the bootstrap-estimated attenuation ratio was 0.92, with a 95% CI spanning 1.0 (0.68–1.27; Supplementary Figure S2, Supplementary Table S1), indicating that the magnitude difference was not robustly supported. These analyses show that *LMOD1* knockdown retains a substantial oxLDL-associated gene-targeting response. In contrast, the angle between the two response vectors was nearly orthogonal (θ = 87.5°, cosine similarity ≈ 0.04), indicating that *LMOD1* knockdown does not simply attenuate (θ ≈ 0°) or invert (θ ≈ 180°) the control program but redirects it along a distinct axis in gene-targeting space (Fig. 4d, Supplementary Table S1). Bootstrap resampling reproduced this estimate (mean θ = 88.3°, 95% CI 83.6–92.1°; Supplementary Fig. 2b, Supplementary Table S1), supporting response redirection rather than simple scaling. A gene-label permutation test confirmed that this angle was significantly smaller than expected for unrelated high-dimensional responses (null mean 90.0°; permutation *p* = 1 × 10^−4^); Supplementary Table S1), indicating that the si*LMOD1* response retains detectable gene-wise structure shared with the control response while being reoriented well away from collinearity; the same result held after gene-wise z-scoring (θ = 87.0°, *p* = 1 × 10^−4^). The same geometry was preserved after gene-wise *z*-scoring of targeting values (θ(z) = 87.0°; attenuation ratio 0.84), indicating that these findings were not driven by differences in inter-gene scale (Supplementary Figure S2, Supplementary Table S1).

Finally, to summarize global separation between condition-level regulatory states, we tested whether the four conditions occupy statistically distinguishable regions of per-sample gene-targeting space using PERMANOVA (Permutational Multivariate Analysis of Variance) on sample-wise dissimilarities (1 − Pearson *r* across genes, after gene-wise *z*-scoring). The omnibus test was significant (R^2^ = 0.41, F = 3.71, *p* = 0.0002), and all six pairwise contrasts remained significant after Holm correction (adjusted *p*-value ≤ 0.043; Supplementary Table S1). Classical multidimensional scaling (MDS) of the condition-level disparity matrix provided a visual summary of this structure (Fig. 4e). The siCON → siCON + oxLDL transition followed a longer trajectory away from baseline, whereas the si*LMOD1* → si*LMOD1* + oxLDL transition was shorter and oriented along a distinct axis, consistent with the vector-angle analysis (Fig. 4d). Because only four condition means are embedded, the MDS in Fig. 4e is used as a visual summary of the pairwise disparities rather than as a fitted model. The two-dimensional embedding captured the dominant structure of the disparity matrix (2D goodness-of-fit = 0.81, with full diagnostics in Supplementary Table S1). Collectively, these results reveal that *LMOD1* deficiency disrupts coordinated oxLDL-induced gene targeting shifts at the per-gene level while redirecting the overall trajectory of the network response, consistent with a reprogrammed gene regulatory state.

### 5. Interaction term analysis distinguishes LMOD1-dependent oxLDL gene-targeting changes

**Figure 5:**
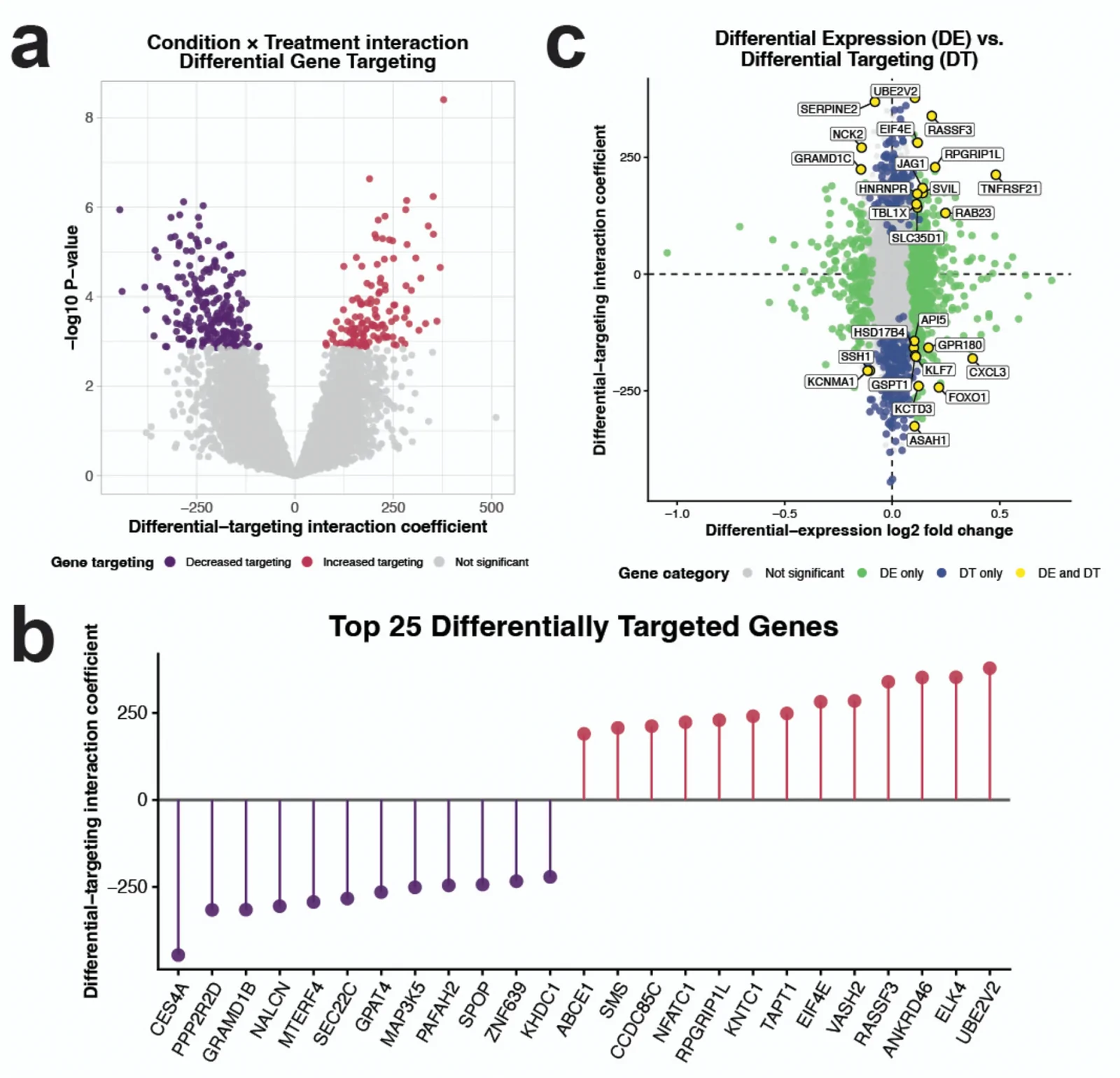
*LMOD1* loss redirects interaction-associated gene targeting and decouples regulatory input from transcriptional output. **(a)** Volcano plot showing genes with significant *LMOD1*-dependent oxLDL interaction effects on inferred gene targeting. The x-axis represents the differential-targeting coefficient, and the y-axis represents −log10 *p*-value. Genes with significantly increased and decreased targeting are shown in pink and purple, respectively. Non-significant genes are shown in gray. **(b)** Lollipop plot showing the top 25 differentially targeted genes ranked by adjusted *p*-value, with ties broken by the absolute differential-targeting coefficient. Each point represents the interaction-associated differential-targeting coefficient for an individual gene. Point color indicates increased or decreased targeting. **(c)** Scatter plot comparing interaction differential expression log2 fold change with differential-targeting coefficient for all genes present in both analyses. Genes significant only in the differential expression analysis are shown in green, genes significant only in the differential targeting analysis are shown in blue, and genes significant in both analyses are shown in yellow and labeled. Genes not significant in either analysis are shown in gray (significant = adjusted *p*-value < 0.05).

Building on the state-level analyses in Fig. 4, we next examined the Condition × Treatment interaction to identify gene-level regulatory programs specifically altered by *LMOD1* deficiency. The interaction model identified a broad set of genes with significant *LMOD1*-dependent changes in inferred regulatory targeting following oxLDL treatment (Fig. 5a). The interaction volcano plot revealed both increases and decreases in inferred interaction-sensitive gene targeting, further supporting the notion that *LMOD1* loss did not simply amplify or suppress the global oxLDL response but instead redirected regulatory targeting in both directions.

To highlight the strongest gene-targeting interaction effects, we visualized the top 25 differentially targeted genes ranked by adjusted *p*-value (Fig. 5b). Some of these genes, including *NFATC1* and *MAP3K5*, are linked to pathways with established roles in SMC phenotypic regulation, namely NFAT-associated vascular SMC proliferation and MAP3K5-dependent oxidative stress signaling[40,41]. However, many of the remaining top-ranked genes are less well annotated in the context of SMC lipid-loading or vascular disease. Because the analysis identifies genes with altered inferred regulatory targeting rather than differential gene expression, some of these targets may represent unrecognized or underexplored components of the SMC response to oxLDL. This network-based approach may therefore uncover *LMOD1*-sensitive regulatory targets that are missed by conventional differential expression analysis.

To determine how these changes in regulatory targeting were related to transcriptional output, we next compared differential gene expression and differential gene targeting for the same interaction contrast (Fig. 5c). This analysis revealed that changes in upstream regulatory targeting and downstream transcriptional output were largely decoupled at the individual gene level, though a smaller subset showed both differential expression and differential targeting. The overlap of genes between these groups suggests that a subset of the *LMOD1*-dependent responses may involve coordinated changes in regulatory input and transcript abundance. Conversely, genes showing differential targeting without differential expression may represent changes in inferred regulatory wiring that are not yet reflected at the steady-state mRNA level, whereas genes showing differential expression without differential targeting may be regulated through mechanisms not represented by inferred TF-gene targeting.

Collectively, these findings suggest that *LMOD1* deficiency reshapes the regulatory response to oxLDL through selective rewiring of gene regulatory programs rather than uniform alterations in transcriptional output. This regulatory rewiring is consistent with the redirected network-level shift observed in Fig. 4 and provides a potential mechanistic basis for the foam cell-like phenotype observed in *LMOD1*-deficient SMCs.

### 6. LMOD1 loss redirects oxLDL-associated TF regulatory influence and downstream propagation programs

After identifying genes with *LMOD1*-dependent oxLDL-induced changes in regulatory targeting, we next asked which upstream TF programs might drive these differences. We therefore estimated TF regulatory influence within each condition-specific network and compared the oxLDL-associated shift in TF influence rank between siCON and si*LMOD1* cells. This approach prioritized TFs whose predicted downstream regulatory influence responded differently after oxLDL depending on *LMOD1* status.

**Figure 6:**
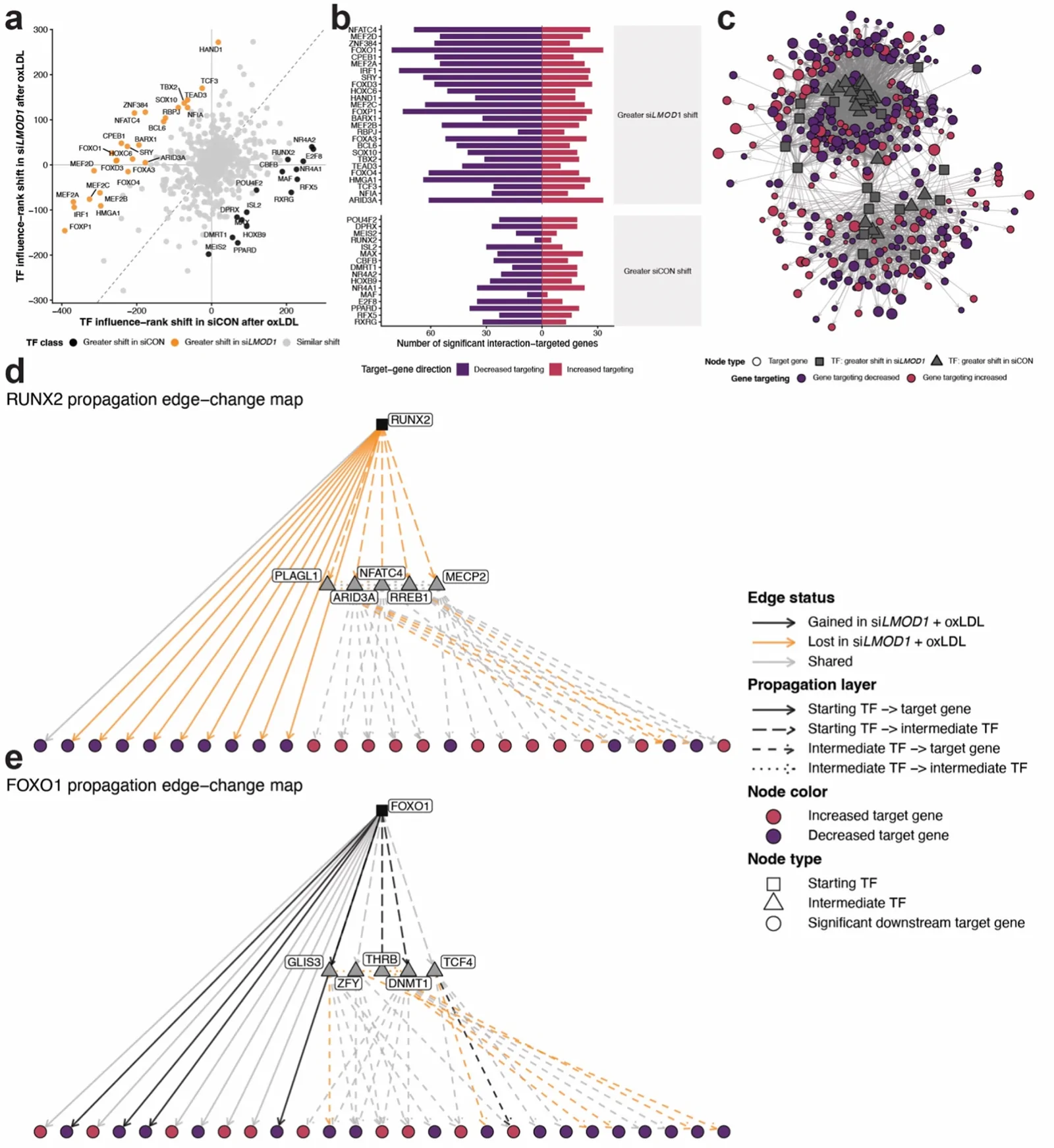
*LMOD1* loss redirects oxLDL-associated TF regulatory influence and downstream propagation programs. **(a)** Scatter plot comparing oxLDL-induced TF regulatory influence-rank shifts in siCON and si*LMOD1* SMCs. Each point represents a TF. The x-axis shows the TF influence-rank shift following oxLDL treatment in siCON, and the y-axis shows the corresponding shift in si*LMOD1*. The dashed diagonal indicates equal oxLDL-associated rank shifts between conditions. TFs are colored according to the direction of their standardized rank interaction effect: orange indicates candidate TFs with positive rank interaction *z*-scores and larger shifts in si*LMOD1*, black indicates candidate TFs with negative rank interaction *z*-scores and larger shifts in siCON, and gray indicates TFs with similar shifts that did not meet the candidate threshold. **(b)** Diverging bar plot summarizing the number of significant *LMOD1*-dependent target genes connected to each candidate TF. Purple bars extending left indicate decreased targeting, whereas pink bars extending right indicate increased targeting. TFs are separated according to whether their oxLDL-induced regulatory influence shift was larger in si*LMOD1* or siCON. **(c)** Network visualization showing all candidate TFs connected to significant *LMOD1*-dependent target genes within the si*LMOD1* + oxLDL regulatory network. TF nodes are shown as squares or triangles, with shape indicating whether the TF showed a stronger oxLDL-induced response in si*LMOD1*or siCON. Target genes are shown as circles and colored by targeting direction. Edges represent top 15% PANDA-inferred TF-to-target regulatory connections. **(d-e)** Propagation edge-change maps for selected candidate TFs: *RUNX2* **(d)** and *FOXO1* **(e)**. For each starting TF, the map displays predicted propagation routes comparing the siCON + oxLDL and si*LMOD1* + oxLDL regulatory networks. Dashed edges represent TF-to-TF regulatory steps, whereas solid edges represent TF-to-target gene regulatory steps. Starting TFs, intermediate TFs, and significant downstream target genes are distinguished by node shape. Target-gene node color indicates increased or decreased *LMOD1*-dependent targeting based on the limma differential targeting analysis.

*LMOD1* loss substantially altered oxLDL-associated TF influence shifts. Most TFs clustered near the center of the TF rank shift scatter plot, indicating similar or only modest rank changes between conditions (Fig. 6a). However, a subset of TFs deviated markedly from the diagonal of equal responses, reflecting pronounced *LMOD1*-dependent differences in oxLDL-induced influence. TFs with positive rank interaction *z*-scores showed larger increases in oxLDL-induced influence shifts in si*LMOD1* cells, whereas those with negative rank interaction *z*-scores exhibited larger shifts in siCON cells. Among the TFs with stronger shifts in si*LMOD1* were *NFATC4*, *FOXO1*, *FOXP1*, *IRF1*, *MEF2A*/*MEF2C*/*MEF2D*, *HMGA1*, *NFIA*, and *ARID3A*, while *RUNX2*, *NR4A1*/*NR4A2*, *PPARD*, *RXRG*, *RFX5*, *E2F8*, and *MAF* showed stronger shifts in siCON cells. Together, these results suggest that loss of *LMOD1* rewires the TF regulatory response to oxLDL, altering the relative influence of key TFs rather than preserving the regulatory response observed in control cells.

We next asked whether these *LMOD1*-sensitive TFs were connected to the downstream genes whose targeting was altered in the interaction analysis. A diverging target-summary plot showed that many candidate TFs were linked to both increased- and decreased-targeting genes identified from the limma interaction differential targeting analysis, indicating that altered TF influence was associated with bidirectional regulatory remodeling (Fig. 6b). These findings suggest that *LMOD1* deficiency affects not only which TFs respond to oxLDL, but also the downstream target-gene programs associated with those TFs.

To visualize these relationships at the network level, we overlaid candidate TFs with significant *LMOD1*-dependent target genes in the si*LMOD1* + oxLDL regulatory network. The resulting network revealed extensive connectivity between candidate TFs and interaction-targeted genes, indicating that *LMOD1* loss affects coordinated TF-target regulatory structure rather than isolated TF or gene changes (Fig. 6c). Target genes exhibiting increased and decreased *LMOD1*-dependent targeting were distributed throughout the network, consistent with broad topological changes between siCON + oxLDL and si*LMOD1* + oxLDL regulatory networks.

Finally, we examined selected TF propagation maps to determine how *LMOD1* loss altered predicted TF-to-TF-to-target routes under oxLDL-treated conditions. *RUNX2* and *FOXO1* were selected as representative examples of distinct TF response patterns. The *RUNX2* propagation map revealed several lost or altered connections in si*LMOD1* + oxLDL relative to siCON + oxLDL, suggesting disruption of the *RUNX2*-associated regulatory route present in control oxLDL-treated cells (Fig. 6d). In contrast, the *FOXO1* propagation map exhibited multiple gained connections in si*LMOD1* + oxLDL, consistent with the stronger *FOXO1*-associated influence shift identified in the si*LMOD1* background (Fig. 6e). Together, these propagation maps illustrate how *LMOD1* loss reshapes the paths through which TFs influence downstream oxLDL-responsive target-gene programs.

Overall, these results indicate that *LMOD1* deficiency also redirects the oxLDL-associated regulatory response at the TF level. Rather than producing a uniform increase or decrease in regulatory activity, *LMOD1* loss is associated with a reorganization of TF influence and downstream propagation through candidate regulators linked to vascular remodeling, inflammation, lipid response, and SMC phenotypic modulation.

## DISCUSSION

This study advances our understanding of how reduced *LMOD1* expression influences SMC phenotypic modulation under atherogenic stress, while also identifying potential mechanisms underlying this process. Using an *in vitro* model of oxidized LDL exposure, we demonstrate that *LMOD1* knockdown in human coronary artery SMCs results in significantly greater intracellular lipid accumulation. Transcriptomic analyses further revealed that *LMOD1* deficiency broadly alters the SMC response to oxLDL, with interaction-associated genes enriched for inflammatory, cytokine-signaling, lipid/atherosclerosis, and vascular disease-related pathways, rather than reflecting a single isolated lipid-handling program. Specifically, we identified a set of genes whose response to oxLDL was modified by *LMOD1* loss, including *LDLR* and *BMP2*, which were validated *in vitro*. At the gene network level, *LMOD1* knockdown redirected the regulatory response to oxLDL, shifting SMCs toward an alternate regulatory state marked by broad differential gene targeting. Finally, TF cascade analysis suggested that this redirected response propagates through altered TF regulatory influence, identifying candidate TF programs whose oxLDL-associated activity differed according to *LMOD1* status. Together, these findings suggest that *LMOD1* may serve as a regulatory buffer that helps stabilize SMC transcriptional and regulatory networks during lipid stress. In its absence, SMCs exhibit greater lipid accumulation and adopt an altered regulatory program. These findings identify *LMOD1* as an important determinant of SMC adaptation to lipid stress and suggest that its loss promotes maladaptive transcriptional remodeling associated with foam cell-like formation.

Our findings add a new dimension to the growing appreciation of SMC contributions to atherosclerosis. It is increasingly recognized that a substantial fraction of foam cells in advanced atherosclerotic lesions is derived from SMCs[7–9]. Consistent with this, *LMOD1* expression is reported to be reduced in human atherosclerotic plaques, potentially predisposing plaque SMCs to accumulate lipid[25]. By demonstrating that *LMOD1* loss promotes lipid accumulation, our study provides a mechanistic explanation for how reduced *LMOD1* expression may contribute to the emergence of a proatherogenic foam cell-like phenotype. This interpretation is consistent with our recent collaborative study, which linked *Lmod1* deficiency to accelerated atherosclerosis and SMC-to-foam-cell transition[24]. However, our previous vascular injury study showed that *LMOD1* deficiency also accelerates SMC proliferation and lesion growth, suggesting that multiple SMC-derived lineages may contribute to disease progression when LMOD1 is reduced[42]. Taken together, these data suggest that LMOD1 is a multifaceted regulator of SMC phenotype, limiting excessive proliferation in response to mechanical injury while also protecting against lipid accumulation and maladaptive transcriptional remodeling in response to oxidative lipid stress. Notably, our findings align with previous reports that other actin-binding or contractile proteins (e.g., transgelin/SM22α, α-smooth muscle actin (α-SMA)) similarly protect against pathological SMC phenotypic modulation in vascular disease[6]. Collectively, these insights highlight the critical role of the smooth muscle cytoskeletal apparatus in maintaining vascular homeostasis and limiting phenotypic transitions that contribute to atherosclerosis.

One notable finding of this study is the identification of *LDLR and BMP2* as *LMOD1*-sensitive genes validated by qPCR. *LDLR* encodes the LDL receptor, a central mediator of receptor-mediated LDL uptake and cholesterol homeostasis, making its altered regulation consistent with the increased lipid accumulation observed in *LMOD1*-deficient SMCs[27]. In contrast, *BMP2* is a pro-osteogenic, phenotypic modulation-associated factor in vascular SMCs. Prior studies have shown that BMP2 promotes phosphate uptake, phenotypic modulation, and calcification in human vascular SMCs[28]. Together, the validation of *LDLR* and *BMP2* suggests that *LMOD1* deficiency affects both lipid-handling and disease-associated phenotypic remodeling pathways during oxLDL exposure. Future studies should determine whether *LMOD1*-mediated changes in *LDLR* expression contribute directly to cholesterol accumulation and whether BMP2 induction reflects a broader shift toward osteogenic or stress-associated SMC phenotypes.

Our network-level analysis provides additional insight into the systems biology of this phenotype. In control SMCs, oxLDL exposure elicited broad changes in inferred gene regulatory interactions, with many genes gaining or losing TF targeting, indicative of extensive transcriptional network remodeling in response to lipid stress. Rather than simply attenuating this response, *LMOD1* knockdown appeared to redirect it. Vector-based analyses indicated that the control and *LMOD1*-knockdown oxLDL responses were nearly orthogonal, suggesting a marked shift in response direction. However, bootstrap estimates did not support a definitive reduction in overall response magnitude. One interpretation is that *LMOD1* helps shape the direction and coordination of the SMC transcriptional response to an atherogenic stimulus. *LMOD1* may also influence key regulators or co-factors that orchestrate gene network shifts through its role as an actin-binding protein and, consequently, through the myocardin-related TF (MRTF)/SRF signaling axis[43,44]. Actin dynamics regulate MRTF nuclear localization and SRF-dependent transcription, which are central determinants of the genes governing SMC phenotype[14,43–45]. *LMOD1* loss could therefore disrupt actin filament homeostasis, impair MRTF/SRF signaling or other mechanotransduction pathways, and thereby alter the regulatory programs engaged during oxLDL exposure, although this possibility requires direct experimental validation. In essence, the loss of *LMOD1* redirects SMCs toward an alternative regulatory state that differs substantially from the canonical lipid-induced remodeling. The comparison of differential expression and differential targeting further suggests that *LMOD1* loss affects both transcript abundance and inferred regulatory control, with only a subset of genes overlapping between these layers. This previously unrecognized role of *LMOD1* in shaping global network responses broadens its influence beyond regulation of individual genes to coordinated control of gene regulatory programs.

While our study sheds light on a novel function of LMOD1 in SMC lipid handling and network regulation, several limitations should be acknowledged. First, our experiments were conducted *in vitro* using cultured human coronary SMCs and acute oxLDL exposure. This approach enabled us to isolate SMC-specific effects, but it cannot fully capture the complexity of atherosclerotic lesions *in vivo*. In actual plaques, SMCs interact with endothelial cells, macrophages, and other immune cells in a milieu of chronic hyperlipidemia and inflammation, which are factors that were not replicated in our cell culture system[5]. Relatedly, although *LMOD1*-deficient SMCs showed increased DiI-oxLDL uptake, we did not consistently detect corresponding changes in macrophage-like markers such as *CD36* and *CD68*. This may reflect the timeline we used for oxLDL exposure and/or donor-specific effects inherent to the use of a single primary SMC donor. Second, our omics analyses (RNA-seq and network inference) were performed on relatively small sample sizes (n = 5 per condition), which may limit statistical power to detect all but the most robust changes. Third, PANDA/LIONESS analyses infer regulatory relationships from gene expression data, TF motif information, and PPI priors; inferred targeting changes should therefore not be interpreted as direct evidence of TF binding or causal regulation. These network findings will require additional validation using approaches such as chromatin accessibility profiling, ChIP-based assays, or targeted perturbation of candidate regulators.

In conclusion, our study demonstrates that the loss of *LMOD1* substantially alters how vascular SMCs respond to atherogenic lipid stress. *LMOD1*-deficient SMCs are driven toward a lipid-laden state, accumulating excess lipid, while mounting a redirected, rather than canonical, transcriptional and regulatory response to oxLDL. Importantly, these findings extend the influence of *LMOD1* beyond its established role in controlling SMC proliferation, identifying it as an important regulator of SMC regulatory homeostasis across distinct pathological stimuli. By linking a smooth muscle cytoskeletal protein to lipid handling and gene network stability, our study provides new insight into the mechanisms governing SMC plasticity in atherosclerosis. From a therapeutic perspective, our work suggests that preserving *LMOD1* expression or downstream functionality in SMCs may represent a strategy to limit plaque progression by reducing SMC-derived foam cell formation and maladaptive phenotypic modulation. Such approaches could ultimately lead to improved therapies for atherosclerotic CVD, which remains a leading cause of mortality worldwide.

## MATERIALS AND METHODS

### Cell culture and treatments

Primary human coronary artery SMCs (HCASMCs, hereafter referred to as SMCs, PromoCell) were cultured in SMC growth medium (Cell Applications) as previously described[46]. All experiments were performed using SMCs at passage 6–8 to ensure consistency in gene expression and cell physiology. Briefly, SMCs were plated in Human SMC Growth Medium (SMGM, Cell Applications #311–500) and transfected the following day with either a non-silencing control siRNA (siCON, Cat# 4390843) or an *LMOD1*-targeting siRNA (si*LMOD1*, Assay ID# s24515) using Lipofectamine RNAiMax (Thermo Fisher Scientific) as previously described[42]. Sixteen hours post-transfection, cells were serum starved for an additional 48 hours and subsequently treated with DiI-labeled oxLDL (Cat# L34358, Thermo Fisher Scientific) for 2 hours. Next, cells were fixed in 4% paraformaldehyde and thereafter counterstained with DAPI. Images were obtained using a fluorescent microscope (Thermo Fisher Scientific EVOS M5000 Imaging System, Invitrogen). The DiI-oxLDL intensity and DAPI-positive cells were quantified using the Celeste 5.0.3 image analysis software (Thermo Fisher Scientific). To account for background fluorescence, a nearby region without staining was selected, and its mean background intensity was recorded. All analyses were performed in a blinded fashion. In some instances, transfected SMCs were treated with 50 μg/mL oxLDL (Cat# L34357, Thermo Fisher Scientific) for 24 hours, after which total RNA was then harvested for mRNA analysis.

### RNA isolation, cDNA synthesis, and quantitative RT-PCR

Total RNA was extracted from cultured cells using the miRNeasy kit (Qiagen), according to the manufacturer’s instructions. Isolated RNA was eluted in RNase-free water and quantified using a NanoDrop spectrophotometer (Thermo Fisher Scientific). Next, cDNA was synthesized using a cDNA synthesis kit (Thermo Fisher Scientific). mRNA levels of *LMOD1, LDLR*, *BMP2*, and *β-Actin* (*ACTB*, used as an internal control) were then assessed using the QuantStudio3 RT-PCR system (Thermo Fisher Scientific). Gene expression data were normalized to *ACTB* and plotted using R. Details of the probes used for gene expression analysis are provided in Supplementary Table S2.

### RNA sequencing and analysis

RNA sequencing was performed by Novogene, resulting in paired-end FASTQ files. Raw reads were quality-assessed with FastQC and aligned to the human reference genome (GRCh38 primary assembly) using STAR (version 2.7.3a) with a genome index built from GENCODE v49 annotations[47,48]. Alignments were run in two-pass mode to improve splice-junction discovery, producing coordinate-sorted BAM files and per-sample, per-gene read counts. BAM files were indexed, and alignment statistics were summarized with SAMtools (version 1.12)[49]. STAR’s --quantMode GeneCounts option produced a per-sample ReadsPerGene.out.tab file for each sample containing four columns: gene ID, unstranded counts, forward-stranded counts, and reverse-stranded counts. Novogene library preparation was confirmed as unstranded.

The per-sample ReadsPerGene.out.tab files were imported into R (v4.5.2) for processing. Gene-level counts were taken from column 2 (unstranded) of each file and assembled into a sample × gene matrix, and Ensembl version suffixes were stripped to base gene IDs. Count matrices were converted to DESeq2 objects via the DESeq2 package (version 1.50.2) after removing genes with ≤1 total count, and size factors were estimated with the median-of-ratios method to normalize for sequencing depth[50]. Differential expression was modeled with a negative-binomial generalized linear model and tested with the Wald statistic; multiple testing was controlled by Benjamini-Hochberg (BH) false discovery rate (FDR) [51]. Log2 fold-changes were shrunk with lfcShrink (type = “ashr”)[52]. Experimental groups included SMCs transfected with either siCON or si*LMOD1* and cultured in the absence or presence of oxLDL (Condition: siCON vs. si*LMOD1*; Treatment: no oxLDL vs. oxLDL). Differential expression was modeled with an interaction design (~ Condition × Treatment) to isolate *LMOD1*-dependent responses to oxLDL. The coefficient ConditionsiLMOD1.TreatmentyesLDL tested whether the oxLDL effect differed between si*LMOD1* and siCON. Significant genes were defined at adjusted *p*-value < 0.05. Quality control and exploratory analysis used rlog-transformed counts and principal component analysis (DESeq2 plotPCA) [50]. Genes from the interaction contrast were visualized as a volcano plot of log2FC vs. −log10(*p*-value), colored using a viridis-inspired palette (pink = upregulated, purple = downregulated, gray = not significant); points with adjusted *p*-value < 1 × 10^−20^ were rendered as triangles to keep the y-axis interpretable. The 25 most significantly differentially expressed genes (ranked by adjusted *p*-value, with ties broken by |log2FC|) were displayed as a lollipop-style plot showing each gene’s log2FC. GO:BP enrichment of the strongest-effect interaction genes (|log2FC| > 0.5) was performed using clusterProfiler’s enrichGO with the full DE table as the background universe and org.Hs.eg.db for human annotation[53,54]. The top 10 enriched terms were visualized as a gene × term heatplot using enrichplot, colored by per-gene log2FC on a viridis scale [55,56].

### Gene regulatory network input data

The GRN analysis conducted in our study integrated RNA-seq expression profiles for each experimental group (siCON, siCON + oxLDL, si*LMOD1*, and si*LMOD1* + oxLDL), PPI data sourced from the STRING Database, and TF motif mappings[37,57,58]. Un-normalized count expression data was imported into a Biobase ExpressionSet object (Biobase version 2.70.0)[59]. The ExpressionSet object was then filtered to remove genes with low counts per million (CPM) using the filterLowGenes function from the yarn R package (version 1.36.0)[60]. Next, data were normalized using the qsmooth function from the qsmooth package (version 1.26.0)[61]. The normalized counts were then log-transformed to stabilize the variance. Human PPI data retrieved from the STRING database (version 12.0) contained confidence scores indicating the probability of a PPI [57]. To keep only high-confidence PPIs, raw interaction scores were divided by 1000 and interactions above 0.7 (considered high confidence) were retained, as performed previously[62]. Ensembl IDs were converted to gene symbols using protein metadata (version 12.0) from STRING to align with PANDA and LIONESS analyses[57]. The PANDA-ready motif mappings, previously prepared by the Glass Lab, involved scanning TF-binding domain sequences (i.e., motifs) in gene transcriptional start sites and linking them to their target genes for subsequent gene regulatory network analysis[37,58]. Motif IDs were trimmed to map them to TF information, and the cleaned TF gene names were extracted to create a final motif network.

### Gene regulatory network construction

PANDA and LIONESS were used to construct GRNs for each sample using the netZooPy Python package (version 0.10.0)[63]. Using data prepared as explained above, we generated gene regulatory networks for each experimental group using the PANDA algorithm. Next, sample-specific gene regulatory networks were constructed using the LIONESS algorithm, which generates individual PANDA networks for each sample. LIONESS then predicts networks specific to individual samples through an iterative process [38]. The final networks selected for further analysis included only the genes overlapping both the motif mappings and the gene expression dataset by using modeProcess=‘intersection’[63]. Next, the LIONESS networks for all four experimental groups (siCON, siCON + oxLDL, si*LMOD1*, and si*LMOD1* + oxLDL) were imported into R for subsequent analysis. PANDA and LIONESS edge weights were softplus-transformed (w’ = ln(1 + e^w^)) following Sonawane et al. methodology[64]. Per-condition thresholds were computed from the corresponding PANDA networks as the midpoint between the median transformed weights of motif-supported edges (prior = 1) and non-motif edges (prior = 0), following the logic implemented in netZooR’s pandaToCondorObject function[65]. For each LIONESS sample-specific network, mean edge weights across samples within a condition were softplus-transformed; edges with transformed mean weight ≥ the corresponding PANDA-derived threshold were retained.

### Data and gene-targeting matrices

Per-sample gene-targeting scores were obtained from PANDA/LIONESS outputs, computed as the weighted in-degree over the edges retained by the per-condition PANDA threshold, using softplus-transformed per-sample edge weights[36]. For each condition c∈siCON,siCON+oxLDL,siLMOD1,siLMOD1+oxLDL, we constructed a gene × sample matrix $M_{c}$ using the intersection of genes present in all conditions. The mean targeting vector for each condition was defined in the following equation, where $m_{c,j}$ is a vector of per-gene targeting scores for sample j in condition c (same gene order across all conditions), $n_{c}$ is the number of samples in condition c, $g_{c}$ is the condition-mean targeting vector (average across the $n_{c}$ samples), and Index i represents genes in the common intersection set used for all analyses:

Equation (1). Mean targeting vector

$$g_{c}=\left(\frac{1}{n_{c}}\right)\;*\;\sum_{j=1}^{n_{c}}m_{c,j}$$

This ensured that comparisons across conditions were performed on a consistent gene set.

### Differential targeting

Gene-by-sample matrices were assembled for siCON, siCON + oxLDL, si*LMOD1*, and si*LMOD1* + oxLDL, and restricted to the intersection of genes present across all four groups. Differential targeting analysis was then carried out using the limma package (version 3.66.0) in R[66]. A single linear model was fit to the per-sample in-degree (gene-targeting) matrix using an interaction design (~ Condition × Treatment) where Condition encoded genotype (siCON, si*LMOD1*) and Treatment encoded oxLDL status (noLDL, yesLDL); coefficients were moderated with empirical Bayes shrinkage via eBayes[67]. All three within- and between-genotype contrasts shown in volcano plots were extracted from this same fit using contrasts.fit and topTable from the limma R package; for Fig. 4a (siCON vs. siCON + oxLDL), the TreatmentyesLDL coefficient, which estimates the oxLDL effect within siCON; for Fig. 4b (si*LMOD1* vs. si*LMOD1* + oxLDL), the contrast TreatmentyesLDL + Conditionsi*LMOD1*.TreatmentyesLDL, which estimates the oxLDL effect within si*LMOD1*; and for Fig. 5a, the interaction coefficient Conditionsi*LMOD1*.TreatmentyesLDL, which tests whether the oxLDL response differs between genotypes. For each contrast, limma produced moderated *t*-statistics, fitted differential-targeting coefficients, *p*-values, and BH-adjusted *p*-values. Volcano plots were constructed in ggplot2 (version 4.0.3) with differential-targeting coefficient on the x-axis and −log10(*p*-value) on the y-axis[68]. Genes were colored using colors pulled from the viridis palette: “Gene Targeting Increased” (adjusted *p*-value < 0.05 and differential-targeting coefficient > 0; pink), “Gene Targeting Decreased” (adjusted *p*-value < 0.05 and differential-targeting coefficient < 0; purple), and “Not Significant” (gray). The 25 most significantly differentially targeted genes were ranked by adjusted *p*-value, with ties broken by the absolute differential-targeting coefficient. These genes were visualized in a lollipop-style plot showing the interaction-associated differential-targeting coefficient gene-targeting log2FC for each gene, with point color indicating increased or decreased targeting. To directly compare *LMOD1*-dependent changes in both gene expression and gene targeting, differential expression and differential targeting interaction results were matched by Ensembl gene ID. Differential expression log2 fold changes were plotted against differential-targeting coefficients, and genes were classified as significant for differential expression (DE), differential targeting (DT), both, or neither based on their adjusted *p*-value < 0.05. Genes significant in both analyses were labeled by gene symbol.

### Targeted boxplots using siCON-defined gene sets

To determine whether *LMOD1* deficiency altered the regulatory response to oxLDL, we first identified two gene sets based on significant targeting changes in siCON vs. siCON + oxLDL. Specifically, genes with a BH-adjusted *p* < 0.05 and a positive or negative *t*-statistic in the siCON vs. siCON + oxLDL contrast were categorized as having significantly increased or decreased targeting, respectively. These two gene sets (419 genes with increased targeting and 387 genes with decreased targeting) were then used as reference groups for further comparison, as done previously[39]. For each gene set, we then extracted the corresponding gene-wise limma *t*-statistics from the si*LMOD1* vs. si*LMOD1* + oxLDL contrast. This enabled direct comparison of oxLDL-induced targeting shifts across genotypes.

All statistical comparisons were performed in R, and visualization was carried out using the ggplot2 package[68]. Boxplots were generated for each gene group, showing the distribution of *t*-statistics per genotype, with a dashed line at *t* = 0 to indicate no change in gene targeting. The primary comparison used paired one-sided Wilcoxon signed-rank tests on per-gene *t*-differences (paired by gene): for the “increased” set, we tested siCON > si*LMOD1* (alternative = “greater”), and for the “decreased” set, we tested siCON < si*LMOD1* (alternative = “less”). We report the matched-pairs rank-biserial effect size r with 95% CIs (via rstatix::wilcox_effsize)[69]. Because the two boxplots are prespecified follow-ups on siCON-defined sets, we present exact *p*-values and emphasize effect sizes and their CIs as the interpretable summary of magnitude and direction. As a gene-set-level robustness check that accounts for inter-gene correlation, we applied CAMERA (limma) within each genotype (baseline vs. oxLDL) using the siCON-defined “increased” and “decreased” sets[66,70]. CAMERA estimates average inter-gene correlation and adjusts the test statistic accordingly. Because the reference sets are siCON-defined, the paired *t*-statistic comparison in Fig. 4c is presented as a descriptive, set-level summary rather than an independent significance test. The genotype dependence of the oxLDL response is assessed formally via the interaction contrast (Fig. 5a).

### Change vectors

Per-sample gene-targeting vectors were obtained from PANDA/LIONESS as described above. For each condition c∈(siCON,siCON+oxLDL,siLMOD1,siLMOD1+oxLDL), we computed the condition-mean targeting vector $g_{c}$ by averaging across its $n_{c}$ sample vectors (Eq. (1))[71]. All operations were performed on the same ordered intersection of genes present in every condition; genes with missing values in any condition were excluded to maintain a common universe.

To quantify the oxLDL response within each genotype, we formed the change vectors by subtracting the baseline mean from the oxLDL mean:

Equation (2). Change vectors

$$\Delta_{\;CON}=g_{CON+oxLDL}-g_{CON}\;\Delta_{\;KD}=g_{KD+oxLDL}-g_{KD}$$

Each entry of Δ is thus the gene-wise mean change in targeting induced by oxLDL within that genotype. These vectors are the inputs for the magnitude (Eq. (3)) and angle (Eq. (4)) analyses.

As a scale-robustness check, we also computed *z*-scored change vectors by standardizing each gene within genotype (mean and s.d. pooled over baseline and oxLDL samples), then repeating the averaging and subtraction to obtain $\Delta^{\left(z\right)}$[72]. This did not alter qualitative conclusions (see Results).

### Response magnitude and attenuation ratio

For each genotype, we quantified the overall size of the oxLDL response as the Euclidean norm of its change vector (Eq. (2)). Given $\Delta_{CON}$ and $\Delta_{KD}$, we computed the magnitudes ‖Δ‖ gene-wise and summarized the attenuation ratio ρ (see below)[71]. To obtain uncertainty, we performed bootstrap resampling within condition (resample samples with replacement; B = 2,000 draws), recomputed Δ, $\|\Delta {\|}_{2}$, and each time, and reported 95% quantile CIs[73]. As a scale-robustness check, we repeated the analysis after gene-wise *z*-scoring within genotype before forming $\Delta^{\left(z\right)}$; conclusions were unchanged (see Results)[72].

Equation (3). Euclidean magnitude and attenuation ratio

$$\|\Delta {\|}_{2}=\sqrt{\sum_{i}\Delta_{i}^{2}}\;\rho =\frac{{\left\|\Delta_{\;KD}\right\|}_{2}}{{\left\|\Delta_{\;CON}\right\|}_{2}}$$

### Angle between responses

We then quantified the alignment between control and knockdown response vectors by computing their angle:

Equation (4). Angle between responses

$$\theta ={cos}^{-1}\left(\frac{\Delta_{\;CON}∙\;\Delta_{\;KD}}{{\left\|\Delta_{\;CON}\right\|}_{2}{\left\|\Delta_{\;KD}\right\|}_{2}}\right)\left(\frac{180}{\pi }\right)$$

To quantify whether LMOD1 knockdown redirects (rather than merely scales) the oxLDL program, we computed the angle θ between the control and knockdown response vectors using the cosine rule with norms from Eq. (3), clamping the cosine argument to [–1, 1] for numerical stability and reporting θ in degrees[74,75]. Uncertainty was obtained by the same within-condition bootstrap ( B = 2000) used for Eq. (3), recomputing θ on each draw and reporting 95% quantile CIs[73,75]. The paired response magnitudes ${\left\|\Delta_{CON}\right\|}_{2}$, ${\left\|\Delta_{KD}\right\|}_{2}$, the attenuation ratio ρ used alongside θ, and the full bootstrap distribution of θ and its confidence interval are shown in Supplementary Figure S2. To assess scale robustness, we recomputed θ using *z*-scored change vectors $\Delta^{\left(z\right)}$ (gene-wise standardization within genotype before forming Eq. (2)), which yielded qualitatively unchanged angles. The *z*-scored response-vector analysis is shown in Supplementary Figure S2.

To test whether the observed inter-vector angle was smaller than expected for unrelated high-dimensional responses, we compared it against a permutation null. We permuted the gene labels of one change (B = 10,000 permutations), which preserves each vector’s Euclidean norm and marginal value distribution while destroying the gene-to-gene correspondence between the two responses, and recomputed θ on each draw. We report the observed angle, the null mean and 95% interval, and a one-sided permutation *p*-value (proportion of null angles < observed). The result was confirmed with a sign-flip null and with gene-wise *z*-scored change vectors.

To compare condition means directly, we defined endpoint disparity between two states a, b as one minus the Pearson correlation of their mean targeting vectors (Eq. (5)), where r is computed across the common gene set and $g_{a}$, $g_{b}$ are condition-mean vectors (Eq. (1)). We computed all pairwise disparities and assembled them into a symmetric 4×4 matrix B=[b(a,b)] (zeros on the diagonal), which served as the dissimilarity input for the embedding described below[74].

Equation (5). Endpoint disparity

$$b(a,b)=1-r\left(g_{a},g_{b}\right)$$

We used the disparity matrix B as the dissimilarity input for classical multidimensional scaling (cmdscale, k = 2) to obtain a 2D embedding of the four condition means[76]. Goodness-of-fit was quantified by the fraction of variance explained by the first two eigenvalues, the RMSE between input and embedded distances, and SMACOF stress[77,78]. Arrows connect baseline → oxLDL within each genotype to illustrate directional transitions. MDS diagnostics, including eigenvalues, goodness-of-fit metrics, strain RMSE, and SMACOF stress, are provided in Supplementary Table S1.

## Pathway enrichment analysis and visualization

Functional enrichment of the significant differentially expressed genes was performed using two approaches. Disease Ontology enrichment was carried out with the EnrichDO R package using doEnrich() and applied to the Entrez IDs of significant DE genes filtered by a |log2FC| > 0.25 threshold[79]. KEGG pathway enrichment was performed with clusterProfiler’s enrichKEGG on the same gene set, using BH adjustment with both *p*-value and q-value cutoffs of 0.05[53,80–82]. For visualization, results from both sources were combined into a single table, and the top 15 terms per source (selected by adjusted *p*-value) were combined into a single-faceted dot plot rendered with ggplot2[68]. Within each facet, terms were ordered along the y-axis by gene ratio. Dot color encodes adjusted *p*-value on a log10-transformed viridis scale, dot size encodes the number of overlapping genes, and the x-axis shows gene ratio[56].

To further characterize the biological processes affected by the strongest-effect DE genes, we performed GO:BP enrichment with clusterProfiler’s enrichGO on significant DE genes filtered by a more stringent |log2FC| > 0.5 cutoff to focus on the strongest-effect genes, using all genes in the DE table as the background universe[53]. The top 10 enriched GO:BP terms were visualized as a heatplot with enrichplot::heatplot, in which rows correspond to terms, columns to member genes, and tile fill encodes each gene’s log2FC on a viridis color scale[55,56].

## TF regulatory influence and propagation analysis

TF regulatory influence analysis was performed using condition-specific PANDA networks generated for siCON, siCON + oxLDL, si*LMOD1*, and si*LMOD1* + oxLDL. This analysis was conceptually adapted from a recently described regulatory network-based framework that used TF targeting and downstream network propagation to quantify inferred TF regulatory influence across biological conditions and identify candidate TFs associated with altered regulatory programs[83]. Here, this framework was applied to identify TFs whose oxLDL-associated regulatory influence differed according to *LMOD1* knockdown status.

Condition-specific PANDA networks were converted from TF-by-gene matrices into TF-to-gene regulatory edge lists, with each row corresponding to a source TF, target gene, and inferred regulatory edge weight. Edge weights were transformed using a softplus function, (w’ = ln(1 + e^w^)), to place weights on a positive scale while preserving relative edge-weight differences, consistent with prior PANDA/LIONESS network processing approaches[64]. Within each condition-specific network, the top 15% of transformed edges were retained to focus the propagation analysis on the strongest inferred regulatory relationships, as done previously[83]. Retained edge weights were then rescaled to bounded, probability-like transition strengths by dividing each edge by the maximum retained edge weight within that condition and multiplying by a probability cap of 0.99, following the general use of weighted adjacency matrices to define transition strengths in network propagation and random-walk models[84]. These values were used to place edges on a relative scale suitable for network propagation and were not intended to represent experimentally measured regulatory probabilities.

For each filtered condition-specific network, TF regulatory influence was estimated by propagating signal through the directed TF-to-target network. The retained edge list was converted into a sparse transition matrix in which rows represented source TFs and columns represented downstream target genes or downstream intermediate TFs. Propagation was performed through the network to a depth of six steps, and the propagated signal was summed across all steps to generate one cumulative downstream regulatory influence score for each TF. This score captures both direct TF-to-target connections and indirect regulatory spread through multi-step paths, such as TF → TF → target gene, thereby estimating the predicted coverage of each TF’s downstream regulatory program within each condition.

To identify TFs whose oxLDL-associated influence depended on *LMOD1* status, TF influence scores were ranked within each condition. For each TF, the oxLDL-associated rank shift was calculated separately within each genetic background as the difference between its oxLDL-treated rank and its corresponding baseline rank. Specifically, the siCON rank shift was defined as rank(siCON + oxLDL) - rank(siCON), and the si*LMOD1* rank shift was defined as rank(si*LMOD1* + oxLDL) - rank(si*LMOD1*). An interaction-style rank shift was then calculated as the difference between these two background-specific shifts: [rank shift in si*LMOD1*] - [rank shift in siCON]. Rank interaction values were *z*-scored across TFs, and TFs with an absolute rank interaction *z*-score greater than 2 were classified as TFs with *LMOD1*-dependent regulatory influence shifts. Positive rank interaction *z*-scores indicated greater oxLDL-associated TF influence shifts in si*LMOD1* relative to siCON, whereas negative values indicated greater shifts in siCON relative to si*LMOD1*.

The oxLDL-associated TF influence-rank shift value in siCON was plotted against the corresponding shift value in si*LMOD1* for each TF. Each point represents one TF, and a dashed diagonal line indicates equal oxLDL-associated influence-rank shifts between siCON and si*LMOD1*. TFs were colored according to the direction of their standardized rank interaction effect: orange indicates candidate TFs with positive rank interaction *z*-scores and greater shifts in si*LMOD1* relative to siCON, black indicates candidate TFs with negative rank interaction *z*-scores and greater shifts in siCON relative to si*LMOD1*, and gray indicates TFs that did not meet the |z| > 2 candidate threshold.

To relate candidate TFs to downstream *LMOD1*-dependent target-gene programs, significant interaction-targeted genes were obtained from the limma gene-level interaction targeting analysis described above. Genes with BH-adjusted *p*-value < 0.05 were retained, and target-gene direction was assigned according to the sign of the interaction differential-targeting coefficient. Genes with positive coefficients were classified as increased targeting, whereas genes with negative coefficients were classified as decreased targeting. The si*LMOD1* + oxLDL regulatory network was then used to quantify the number of significant interaction-targeted genes connected to each candidate TF. Candidate TF-to-target edges were retained when the source TF was an interaction candidate and the target gene was significant in the interaction gene targeting analysis. For each TF, connected target genes were counted separately by targeting direction. TFs were ordered by rank interaction *z*-score and faceted according to whether their oxLDL-associated influence-rank shift was greater in si*LMOD1* or siCON.

Candidate TFs and significant interaction-targeted genes were also overlaid onto the si*LMOD1* + oxLDL regulatory network to generate a focused TF-target subnetwork. Directed edges were retained when the source node was a candidate TF and the target node was a significant interaction-targeted gene. The resulting graph was constructed using igraph and visualized with ggraph[85,86]. TF nodes were represented as squares or triangles according to whether they had greater oxLDL-associated influence-rank shifts in si*LMOD1* or siCON, respectively. Target genes were represented as circles and colored according to targeting direction. Target-gene node size was scaled according to interaction significance based on the adjusted *p*-value.

Propagation edge-change maps were generated for selected TFs to compare predicted regulatory routes between oxLDL-treated conditions. *RUNX2* and *FOXO1* were selected as representative candidate TFs with distinct oxLDL-associated influence-shift patterns. Edge lists from the two oxLDL-treated networks were joined by source and target node, and each edge was classified as shared, lost in si*LMOD1* + oxLDL, or gained in si*LMOD1* + oxLDL. For each selected starting TF, TF → intermediate TF edges were identified by retaining edges from the starting TF to downstream nodes that were also TFs in the regulatory network. Intermediate TF → significant target-gene edges were then identified by retaining edges from intermediate TFs to significant interaction-targeted genes. These edges were joined to define TF → TF → target gene propagation paths. Paths were scored as the product of the maximum probability-like edge weight for the starting TF-to-intermediate TF edge and the maximum probability-like edge weight for the intermediate TF-to-target gene edge across the two oxLDL-treated networks. The top intermediate TFs were selected based on the number of significant targets reached, and the top downstream targets per intermediate TF were selected by path score. *RUNX2* and *FOXO1* propagation maps were generated using the top 8 intermediate TFs and top 5 downstream targets per intermediate TF, with shared edges retained to visualize conserved propagation structure alongside condition-specific gained or lost edges. Propagation maps were visualized as directed graphs using a layered Sugiyama-like layout. Starting TFs, intermediate TFs, and significant downstream target genes were distinguished by node shape; target-gene node color indicated increased or decreased *LMOD1*-dependent targeting; edge color indicated whether a regulatory connection was gained, lost, or shared; and edge line type distinguished starting TF → intermediate TF and intermediate TF → target gene propagation layers.

## Statistical analysis

Quantitative data were analyzed and visualized in R using ggplot2 for plotting, ggpubr (version 0.6.3) and rstatix (version 0.7.3) for statistical testing, and car (version 3.1–5) for variance tests[68,69,87,88]. Before statistical analyses were performed, outliers were evaluated using rstatix::identify_outliers, with Grubbs’ tests used as an additional check where needed[69]. For two-group outcomes (e.g., DiI-oxLDL uptake), we assessed normality within each group using the Shapiro-Wilk test and homogeneity of variances using Bartlett’s test to characterize the data, then used a variance-robust two-sample Welch’s t-test in the stats base package in R[87]. For multi-group outcomes, we fit a one-way ANOVA to obtain residuals and tested assumptions with Shapiro-Wilk and Levene’s test using the stats and car packages[87,88]. If residuals were approximately normal and variances were equal, we used one-way ANOVA followed by Tukey’s HSD. If normality held but variances were unequal, we used Welch’s ANOVA followed by Games-Howell[69,87]. If normality failed, we used Kruskal-Wallis followed by pairwise Wilcoxon rank-sum post hoc tests and BH adjustment. Box plots show the interquartile range (IQR; 25th −75th percentiles), the median as a horizontal line, and whiskers extending to the most extreme points within 1.5 × IQR[68]. For paired comparisons of per-gene gene-targeting metrics (e.g., matched *t*-statistics across conditions in Fig. 4c), we used the Wilcoxon signed-rank test with directional alternatives, and reported matched-pairs rank-biserial effect sizes with 95% CIs[87]. CAMERA was used as a gene-set-level robustness check, and bootstrap resampling was used to estimate uncertainty for response-vector angles, Euclidean norms, and attenuation ratios[66,70]. PERMANOVA was performed using vegan::adonis2 with Holm-adjusted pairwise contrasts[89]. RNA-seq differential expression was performed with DESeq2[50]. Raw counts were normalized by the median-of-ratios method[50]. A negative binomial GLM with an interaction design (~ Condition × Treatment) was fit per gene, Wald tests provided *p*-values, and multiple testing was controlled by Benjamini-Hochberg FDR[50]. Principal component analysis (PCA) for sample relationships was performed on regularized log-transformed counts[50].

## Supplementary Material

Supplementary Files

This is a list of supplementary files associated with this preprint. Click to download.

• Suppplementarycombined.pdf

## Data Availability

The code required to reproduce all figures and tables in this article are publicly available on GitHub at https://github.com/NandaLab-UAB/NandaLab_Project_2. The RNA-seq data generated in this study have been deposited in the Gene Expression Omnibus (GEO) under accession number GSE342401.
